# Vitamin D supplementation and central adiposity in male shift workers: a randomized clinical trial on metabolic health

**DOI:** 10.1590/1516-3180.2025.2955.18032026

**Published:** 2026-08-21

**Authors:** Luiz Menezes-Júnior, Virgínia Capistrano Fajardo, Fernando Luiz Pereira de Oliveira, George Luiz Lins Machado Coelho, Fausto Aloisio Pedrosa Pimenta, Raimundo Marques do Nascimento, Sílvia Nascimento de Freitas

**Affiliations:** I Professor, Programa de Pós-Graduação em Saúde e Nutrição (PPGSN), Escola de Nutrição (ENUT), Universidade Federal de Ouro Preto (UFOP), Ouro Preto (MG), Brazil.; II Programa de Pós-Graduação em Ciências Aplicadas à Saúde do Adulto, Universidade Federal de Minas Gerais (UFMG), Belo Horizonte (MG), Brazil.; III Associate Professor, Instituto de Ciências Exatas e Biológicas (ICEB), Universidade Federal de Ouro Preto (UFOP), Ouro Preto (MG), Brazil.; IV Associate Professor, Programa de Pós-Graduação em Saúde e Nutrição (PPGSN), Escola de Medicina, Universidade Federal de Ouro Preto (UFOP), Ouro Preto (MG), Brazil.; V Associate Professor, Escola de Medicina, Universidade Federal de Ouro Preto (UFOP), Ouro Preto (MG), Brazil.; VI Associate Professor, Escola de Medicina, Universidade Federal de Ouro Preto (UFOP), Ouro Preto (MG), Brazil.; VII Associate Professor, Programa de Pós-Graduação em Saúde e Nutrição (PPGSN), Escola de Nutrição (ENUT), Universidade Federal de Ouro Preto (UFOP), Ouro Preto (MG), Brazil.

**Keywords:** Shift Work Schedule, Circadian rhythm, Calcitriol, Obesity, Vitamin D, Calciferol, Adiposity, Clinical trial

## Abstract

**BACKGROUND:**

Vitamin D deficiency is common among individuals with obesity; however, the association between low 25-hydroxyvitamin D [25(OH)D] concentrations and adiposity remains unclear. Shift workers are at increased risk for both vitamin D deficiency and metabolic disorders, making this population particularly relevant for investigating this relationship.

**OBJECTIVES:**

This study aimed to evaluate the effect of cholecalciferol supplementation on central adiposity, assessed by waist circumference (WC) and waist-to-height ratio (WHtR), among male shift workers.

**DESIGN AND SETTING:**

Randomized, double-blind, placebo-controlled clinical trial involving 198 male shift workers employed by an iron ore extraction company in Minas Gerais, Brazil.

**METHODS:**

Participants with 25(OH)D concentrations <30 ng/mL and at least one metabolic abnormality, including metabolic syndrome, impaired fasting glucose, dyslipidemia, elevated blood pressure, or increased WC, were randomly assigned to receive placebo, cholecalciferol 14,000 IU/week, or cholecalciferol 28,000 IU/week for 4 months. Plasma 25(OH)D concentrations, WC, and WHtR were evaluated at baseline and post-intervention.

**RESULTS:**

After 4 months, no significant differences in WC or WHtR were observed between the vitamin D supplementation groups (14,000 IU/week and 28,000 IU/week) and the placebo group. Nevertheless, serum 25(OH)D concentrations increased significantly in the supplementation groups: +6.23 ng/mL (p25 = 0.58; p75 = 10.61) in the 14,000 IU/week group and +15.25 ng/mL (p25 = 10.57; p75 = 19.68) in the 28,000 IU/week group. In contrast, the placebo group showed a decrease of −0.50 ng/mL (p25 = −1.03; p75 = 2.71). Despite these increases, supplementation produced no significant effect on central adiposity outcomes (p > 0.05).

**CONCLUSION:**

Although vitamin D supplementation effectively increased serum 25(OH)D concentrations, it did not reduce central adiposity among male shift workers. These findings indicate that vitamin D supplementation alone is unlikely to improve obesity-related outcomes in this high-risk occupational population and underscore the need for comprehensive strategies targeting metabolic health in shift workers.

**CLINICAL TRIAL OR SYSTEMATIC REVIEW REGISTRATION:**

This study was registered in the Brazilian Registry of Clinical Trials (ReBEC) under identifier RBR-6CS352 on October 25, 2017.

## INTRODUCTION

 Obesity is a chronic, multifactorial disease characterized by excessive adipose tissue accumulation and is among the most significant global public health challenges. It contributes to millions of deaths each year and is strongly associated with comorbidities such as hypertension, type 2 diabetes, and cardiovascular disease.^1^ In Brazil, the burden is similarly substantial, with 39.6% of the population classified as overweight and 23.8% as obese.^2^ The rising prevalence of obesity highlights the need to identify modifiable risk factors and develop effective interventions. 

 Vitamin D deficiency has emerged as a potential factor associated with obesity and is now recognized as a worldwide health concern affecting all age groups.^3^ Cross-sectional studies have consistently demonstrated an inverse relationship between serum 25(OH)D concentrations and adiposity, as well as associations with metabolic abnormalities, including insulin resistance, type 2 diabetes, and dyslipidemia.^4^ Experimental evidence suggests that vitamin D may regulate leptin expression, a hormone involved in appetite control and energy homeostasis, indicating a possible role in adipogenesis and obesity-related mechanisms.^5^ Nevertheless, despite these findings, the causal relationship between vitamin D deficiency and obesity remains uncertain.^6,7^ Additional high-quality evidence is needed to clarify the contribution of vitamin D to obesity management and related metabolic disorders. Furthermore, occupational factors have been identified as important determinants of vitamin D status. 

 Shift workers are particularly vulnerable because reduced sunlight exposure limits endogenous vitamin D synthesis. Studies have shown that shift workers have an 80% higher risk of hypovitaminosis D than individuals in other occupational groups.^8^ In this population, Batista et al.^9^ reported a high prevalence of hypovitaminosis D (73%) and a significant association with increased visceral fat area (odds ratio [OR]: 2.4; 95% confidence interval [CI]: 1.1–5.2). These findings highlight the importance of targeted strategies to address vitamin D deficiency among shift workers. 

## OBJECTIVE

This study aimed to evaluate the effect of cholecalciferol supplementation on central adiposity, measured by changes in waist circumference (WC) and waist-to-height ratio (WHtR), among male shift workers.

## METHODS

### Study design and population

 This study was conducted within the *Fatigue Management Project*, an ongoing research program investigating metabolic and occupational health among rotating shift workers in Brazilian mining companies since 2012 through cross-sectional assessments performed in 2012, 2015, and 2018. The present analysis derives from a randomized, double-blind, placebocontrolled clinical trial conducted in 2016 and nested within the *Fatigue Management Project*. The trial was designed to assess the effects of vitamin D supplementation on biochemical and body composition risk factors related to fatigue, cardiovascular disease, and cognitive function.^10^ The intervention was implemented during the summer. 

 The study included male employees of an iron ore mining company located in the Quadrilátero Ferrífero (Iron Quadrangle) region of Minas Gerais, Brazil. Eligible workers were truck drivers and heavy machinery operators who used automated, climatecontrolled equipment and worked rotating shifts. The schedule followed a clockwise rotation (morning, afternoon, and night shifts) consisting of 6 hours of work followed by 12 hours of rest. After four consecutive shifts, workers received 1 day off, resulting in a weekly workload of 36 hours. 

 Sample size calculations were based on the assumptions that hypertriglyceridemia would be present in 40% of participants not receiving vitamin D supplementation and in 22% of those receiving supplementation, with a likelihood ratio of 2.3.^10^ Considering 80% statistical power and a 95% CI, 106 participants were required per group. After accounting for an anticipated 10% attrition rate, the minimum target sample was 350 participants. Sample size estimation was performed using OpenEpi (Open Source Epidemiologic Statistics for Public Health, United States), version 3.01. 

**Figure 1:**
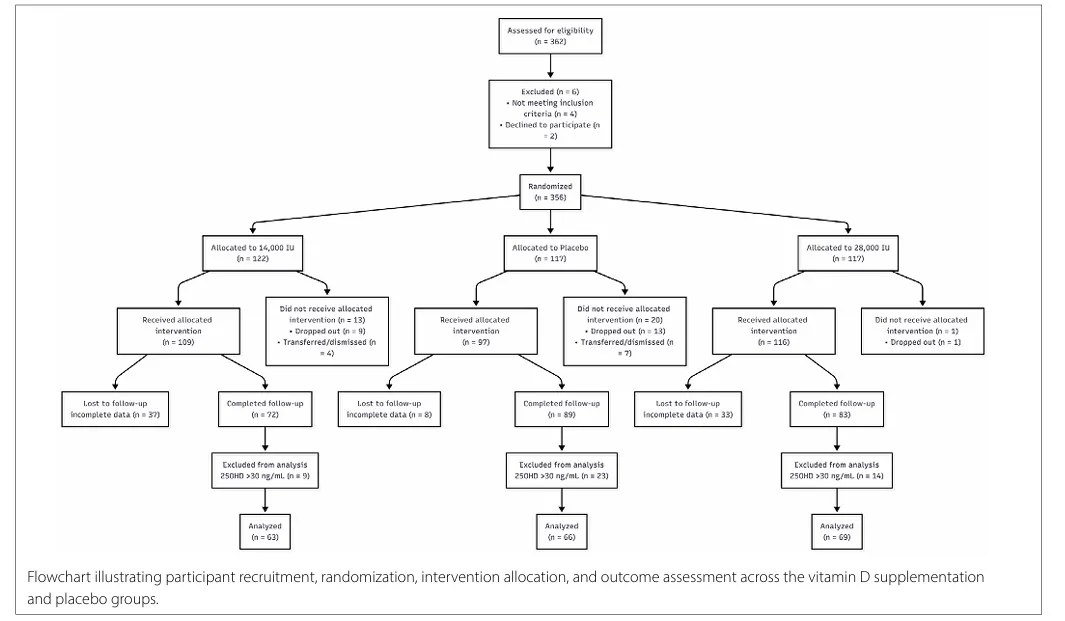
Study flowchart.

 The analysis included participants who completed the 4-month intervention period. Eligibility was restricted to individuals with baseline 25(OH)D concentrations < 30 ng/mL and complete socioeconomic and anthropometric data collected before and after the intervention. A total of 362 workers were initially recruited. Of these, 44 did not receive the intervention because of withdrawal, transfer, or employment termination. Following randomization, 120 additional participants were excluded because their 25(OH)D concentrations exceeded 30 ng/mL or because of incomplete data. Consequently, 198 participants were included in the final analysis (**Figure 1**). 

 According to the study protocol, primary and secondary outcomes were planned for analysis using both intention-to-treat (ITT) and per-protocol (PP) populations.^1^ The ITT population comprised all randomized participants with available outcome data, analyzed according to their assigned groups. The PP population excluded individuals with protocol deviations or those who failed to meet all biochemical inclusion criteria identified after allocation. The present manuscript reports findings from the PP population (n = 198) to evaluate the clinical efficacy of cholecalciferol supplementation among participants with confirmed baseline 25(OH)D insufficiency (< 30 ng/mL). 

 The study was conducted in accordance with the Consolidated Standards of Reporting Trials (CONSORT) guidelines. A completed CONSORT checklist is provided as supplementary material. 

### Intervention

 Participants were randomly assigned to one of three groups: placebo, cholecalciferol 14,000 IU/week, or cholecalciferol 28,000 IU/week. The intervention groups received oral cholecalciferol (vitamin D3) supplementation at the assigned dosage, whereas the placebo group received identical capsules without cholecalciferol. All participants received bottles containing 10 capsules and were instructed to take one capsule weekly. 

 To promote adherence and reduce missed doses, the research team conducted regular follow-up contacts, providing individualized reminders on scheduled administration days. Supplement distribution was coordinated through prearranged workplace visits, during which participants received vitamin D or placebo bottles and brief reinforcement regarding protocol compliance. 

 Blinding procedures were implemented by a study statistician who was not involved in data collection or participant contact. This investigator generated the randomization sequence and assigned participants to the study groups. Placebo and active treatment capsules were identical in appearance and packaging. All capsules were labeled with participant codes to conceal treatment allocation from participants, intervention personnel, and outcome assessors. Group assignments remained masked until completion of data verification and statistical analyses. A posttrial evaluation of participants’ allocation guesses supported the effectiveness of the blinding procedures. 

 Eligibility criteria were established according to the primary objective of the clinical trial, which was to assess the effects of cholecalciferol supplementation on cardiometabolic risk factors. Participants were required to have serum 25(OH)D concentrations < 30 ng/mL and at least one of the following conditions: metabolic syndrome, impaired fasting glucose, dyslipidemia, elevated blood pressure, or increased WC. Exclusion criteria included malabsorption syndrome; renal, hepatic, or thyroid disease; use of anticonvulsants, corticosteroids, hormones, vitamin supplements, or mineral supplements; and abnormal serum concentrations of creatinine, parathyroid hormone, calcium, or albumin. 

 Stratified randomization was used to ensure balanced distribution of vitamin D status and cardiovascular risk factors across the two intervention groups and the placebo group (**Figure 1**), consistent with the primary objectives of the parent clinical trial.^9^

 Adverse events were monitored throughout the study via weekly reminder messages and follow-up assessments conducted at months 4, 8, and 12.. Participants were instructed to report symptoms suggestive of vitamin D toxicity, such as dizziness, nausea, or constipation, as well as any other health-related changes experienced during the trial. 

### Data collection and definitions

 Data were collected between January and July 2016 at five mining facilities operated by the company. Biological samples were processed at the Pilot Clinical Analysis Laboratory (LAPAC) and the Laboratory of Epidemiology of Parasitic Diseases (LEPI) of the Escola de Medicina, Universidade Federal de Ouro Preto (UFOP). A trained research team conducted all study procedures, including administration of sociodemographic questionnaires, anthropometric assessments, and collection and analysis of biological samples. 

 Sociodemographic information was obtained using a standardized questionnaire administered by trained personnel. Variables included sex, age (years), marital status (single, married, separated/divorced, or widowed), and educational attainment (primary, secondary, or higher education). Skin color was self-reported according to the classification adopted by the Brazilian Institute of Geography and Statistics (white, black, brown, yellow, or indigenous). Duration of shift work was determined from the total number of years worked in rotating shifts. 

### ADIPOSITY MARKERS

 WC was measured using a flexible, nonelastic tape positioned midway between the iliac crest and the lowest rib margin.^11^ Participants removed their shirts and stood upright with a relaxed abdomen, arms at their sides, and feet together. Measurements were obtained in triplicate while participants breathed normally to avoid abdominal muscle contraction.^12^ WC was classified according to International Diabetes Federation criteria,^13^ with values ≥ 90 cm considered elevated. Height was measured using a portable AlturExata^®^ stadiometer (AlturExata, Belo Horizonte) with 1-mm precision. The WHtR was calculated by dividing WC by height. WHtR values ≥ 0.5 were classified as elevated.^14^

### Vitamin D

 Serum vitamin D concentrations were determined by chemiluminescence using Beckman Coulter^®^ commercial kits (Beckman Coulter, Fullerton, California, United States). Vitamin D status was classified according to the Brazilian Society of Endocrinology and Metabolism guidelines: deficiency (< 20 ng/mL), insufficiency (20–29 ng/mL), and sufficiency (≥ 30 ng/mL).^15^

### Statistical analysis

 Data analysis was performed using Stata/MP version 15.0 and R version 3.5.0 for Linux (The R Foundation for Statistical Computing). Categorical variables are presented as absolute frequencies (n) and percentages (%). Continuous variables are reported as mean ± standard deviation or median (p25–p75), according to data distribution assessed with the Kolmogorov–Smirnov test. 

 Statistical analyses were conducted in three stages. First, baseline comparability among the three randomized groups was evaluated using Pearson’s chi-square (χ^2^) test. Second, intervention effects after 4 months were assessed using McNemar’s chi-square test for categorical variables and paired Student’s t test or the Wilcoxon signed-rank test for continuous variables, as appropriate. Third, post-intervention differences among groups were examined using the KruskalWallis test followed by Bonferroni-adjusted post hoc comparisons. 

 Changes in WC and WHtR were additionally assessed using percentage variation between baseline and 4 months. Percentage change was calculated as: [(final value-initial value)/final value) × 100]. This measure represented the proportional change relative to the final value. 

 Participants with incomplete outcome data were excluded from the analysis, resulting in a per-protocol approach. Baseline characteristics of completers and non-completers were compared to evaluate potential attrition bias (data not shown). 

### Ethical considerations

 The study protocol was approved by the Research Ethics Committee of the Universidade Federal de Ouro Preto (CEP 1.381.376) on December 17, 2015. The trial was subsequently registered in the Brazilian Registry of Clinical Trials (RBR-6CS352) under the title “Fatigue Management Project.” The study was conducted under the responsibility of the Escola de Medicina, Universidade Federal de Ouro Preto. All participants provided written informed consent prior to enrollment. 

## RESULTS

 The study included 198 rotating shift workers aged 24–57 years. The largest age group was 30–39 years (51.5%), followed by 40–49 years (35.9%). Most participants (93.9%) had worked rotating shifts for >5 years. More than half had completed high school and self-identified as non-White (**Table 1**). The prevalence of abdominal obesity was 72.2% (n = 143) based on WC and 83.3% (n = 165) based on the WHtR (data not shown). 

**Table 1:** Sociodemographic profile of shift workers in a mining company in the Iron Quadrangle region, Minas Gerais

**Variables**		**n**	**%**
Age group			
	*24–39 years*	110	55.5
	*40–57 years*	88	44.5
Years of shift work			
	*<10 years*	71	35.9
	*≥10 years*	121	64.1
Marital status			
	*Married*	167	84.3
	*Single*	31	15.6
Education level			
	*Up to primary school*	7	3.5
	*High school/Technical*	180	90.9
	*Higher education*	11	5.6
Skin color			
	*White*	58	29.3
	*Non-white*	140	70.7

Data represent the sociodemographic profile of shift workers.

 No significant baseline differences were observed between treatment groups for WC (p = 0.487), WHtR (p = 0.973), age (p = 0.563), ethnicity (p = 0.694), shift work duration (p = 0.794), or baseline plasma vitamin D levels (p = 0.083) (**Table 2**). 

**Table 2:** Sociodemographic, anthropometric, and vitamin D characterization of the alternating shift workers sample at baseline

		**Placebo (n = 63)**	**14,000 IU (n = 66)**	**28,000 IU (n = 69)**	**p value**
Age group					
	*24–39 years*	32 (16.2%)	35 (17.7%)	43 (21.6%)	0.363
	*40–57 years*	31 (15.7%)	31 (15.7%)	26 (13.1%)	
Skin color					
	*White*	21 (10.6%)	18 (9.1%)	19 (9.6%)	0.694
	*Non-white*	42 (21.2%)	48 (24.2%)	50 (25.3%)	
Waist circumference					
	*<90 cm*	18 (9.1%)	15 (7.6%)	22 (11.1%)	0.487
	*≥90 cm*	45 (22.7%)	51 (25.8%)	47 (23.7%)	
Waist-to-height ratio					
	*<0.5*	11 (5.6%)	11 (5.6%)	11 (5.6%)	0.973
	*≥0.5*	52 (26.2%)	55 (27.8%)	58 (29.2%)	

Pearson’s chi-square test was used to verify homogeneity between intervention groups.

 Among participants receiving 14,000 IU/week of cholecalciferol, insufficient and deficient vitamin D levels decreased by 16.2% and 3%, respectively, whereas sufficient levels increased by 19.3%. In the 28,000 IU/week group, insufficient and deficient vitamin D levels decreased by 21.8% and 7.1%, respectively, while sufficient levels increased by 28.8%. In contrast, the placebo group exhibited a 1.5% increase in insufficient vitamin D levels, a 3.0% decrease in deficient levels, and a 1.5% increase in sufficient levels between pre- and post-intervention assessments. 

**Figure 2:**
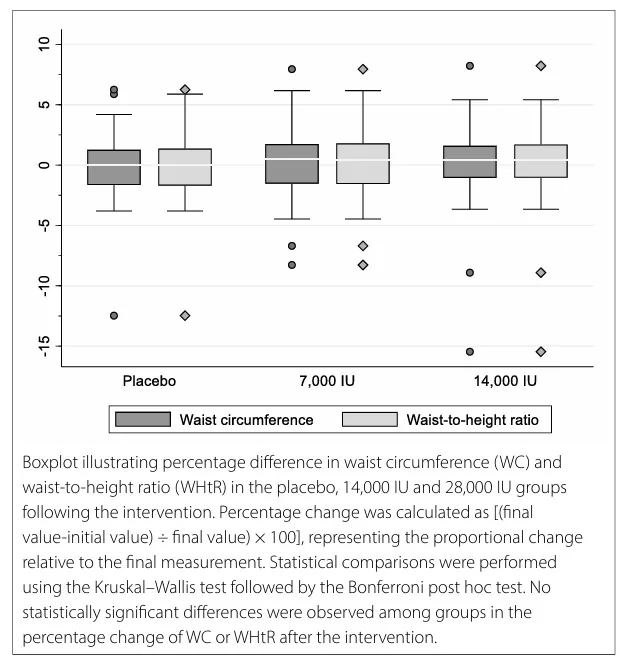
Percentage difference in waist circumference (WC) and waist to-height ratio (WHtR) after the intervention.

 No significant changes in WC or WHtR were detected following supplementation in any intervention group (**Table 3**). However, WC and WHtR adequacy rates increased by 2.5% and 0.5%, respectively, in the 14,000 IU/week vitamin D3 group. In the 28,000 IU/week group, the proportion of participants with adequate WC decreased by 2%, whereas WHtR remained unchanged (**Figure 2**). In the placebo group, WC adequacy decreased by 1%, whereas WHtR adequacy increased by 1% (**Table 3**). 

**Table 3:** Difference in vitamin D levels and waist circumference after 4 months between the intervention and placebo groups.

	**Placebo**	**14,000 IU**	**28,000 IU**
25(OH)D (ng/mL)	−0.5 (−3.73; 2.71)^a^	6.23 (0.58; 10.61)^b^	15.25 (10.57; 19.68)^c^
WC (cm)	−0.1 (−1.8; 1.4)^a^	0.37 (−1.25; 1.6)^a^	0.8 (−1; 1.83)^a^
* WHtR (e^-1^)	0.01 (−1.03; 0.08)^a^	−0.07 (0.02; 0.09)^a^	0.04 (−0.05; 1.03)^a^

Data are presented as median (p25–p75). Comparisons were performed using the Kruskal–Wallis test followed by the Bonferroni post hoc test. Superscript letters (a, b, c) indicate statistically significant differences between groups. Groups sharing the same letter do not differ significantly (p > 0.05), whereas groups with different letters differ significantly (p < 0.05).

* WHtR raised to −1.

 Plasma 25(OH)D levels increased substantially in both supplementation groups. The 14,000 IU/week group demonstrated a median increase of 6.23 ng/mL (p25 = 0.58; p75 = 10.61), whereas the 28,000 IU/week group showed a median increase of 15.25 ng/mL (p25 = 10.57; p75 = 19.68). Conversely, the placebo group exhibited a median decrease of 0.50 ng/mL (p25 = −3.73; p75 = 2.71) in plasma 25(OH)D levels (**Table 3**). 

**Figure 3:**
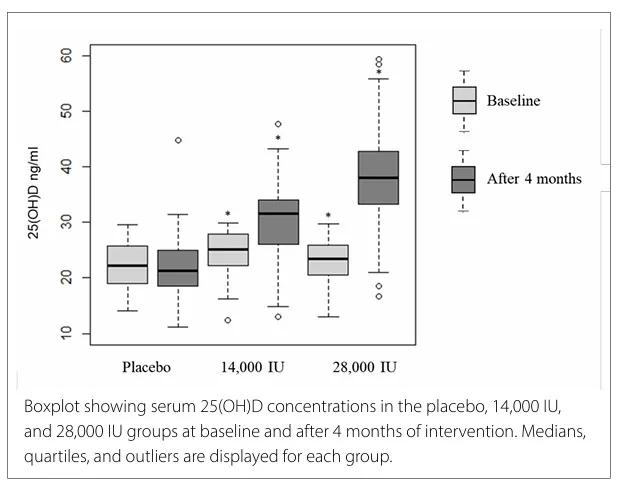
Serum vitamin D [25(OH)D] levels before and after the intervention.

 As illustrated in **Figure 3**, both vitamin D supplementation groups demonstrated significantly greater increases in 25(OH)D levels than the placebo group. No adverse events or unintended effects associated with supplementation were reported or observed during the 4-month follow-up period. 

## DISCUSSION

 This randomized, placebo-controlled trial evaluated the effects of weekly oral cholecalciferol supplementation at doses of 14,000 IU and 28,000 IU for 16 weeks in individuals with baseline 25(OH)D concentrations < 30 ng/mL. Although supplementation significantly increased 25(OH)D levels in both intervention groups (p < 0.05), no significant changes were observed in WC or WHtR compared with placebo. 

 Cross-sectional studies have consistently reported an inverse association between 25(OH)D concentrations and adiposity.^16-18^ In a study of 250 adults in New Zealand, serum vitamin D levels decreased by 0.12 ng/mL for each 1-cm increase in WC (p = 0.01) and by 0.30 ng/mL for each 1 kg/m^2^ increase in body mass index (BMI) (p = 0.002).^19^ However, studies designed to evaluate causality have not produced similar findings. A randomized clinical trial involving 328 participants reported that vitamin D supplementation of up to 4,000 IU/day for 3 months had no significant effect on BMI (mean difference [MD], −0.06 kg/m^2^; 95% CI, −0.14 to 0.03), body weight (MD, −0.05 kg; 95% CI, −0.32 to 0.23), or fat mass (MD, −0.43 kg; 95% CI, −1.69 to 0.84) compared with placebo.^20^ These findings are consistent with the present study. 

 Although BMI, WC, and WHtR are widely used because of their simplicity, low cost, and established associations with disease risk, these measures do not directly quantify body fat. Bioelectrical impedance analysis (BIA) offers a validated alternative for estimating body fat in clinical settings.^21^ In a study of 1,105 adults aged 20–70 years, Zhang et al.^22^ reported an inverse association between serum 25(OH)D concentrations and visceral fat area (VFA) estimated by BIA. Higher VFA was associated with an increased likelihood of vitamin D insufficiency or deficiency, demonstrating a significant dose-response relationship (p < 0.001). 

 Nevertheless, both anthropometric measures and BIA have limited ability to distinguish abdominal adipose tissue from retroperitoneal fat, potentially weakening estimates of the association between vitamin D status and adiposity. More precise assessments can be obtained using dual-energy X-ray absorptiometry (DXA), computed tomography (CT), and magnetic resonance imaging (MRI).^23^ A randomized, double-blind, placebo-controlled trial involving 52 participants aged 18–50 years used DXA and MRI to assess body composition and found that daily supplementation with 7,000 IU of cholecalciferol for 26 weeks significantly increased 25(OH)D concentrations but produced no changes in total body fat, subcutaneous adipose tissue, or visceral fat relative to placebo.^7^

 Obesity is a multifactorial disease influenced by genetic, socioeconomic, cultural, and behavioral determinants.^24^ Consequently, vitamin D supplementation alone may be insufficient to modify obesity-related outcomes. This interpretation is supported by an analysis of 21 adult cohorts showing that higher BMI leads to lower concentrations of 1,25(OH)_2_D, whereas no evidence indicated that vitamin D deficiency contributes to obesity development.^4^ Similarly, randomized controlled trials, including the present study, have not supported a causal effect of vitamin D deficiency on adiposity.^7,20,25,26^

 Physical inactivity is another factor associated with obesity and reduced vitamin D status because it may limit sun exposure. Approximately 90% of vitamin D is synthesized endogenously through exposure to sunlight.^27^ Therefore, conditions that restrict outdoor activity, including sedentary behavior and shift work, may contribute to lower circulating 25(OH)D concentrations. A study of 5,409 workers in South Korea aged 20–65 years reported a significantly greater risk of vitamin D deficiency among shift workers than among daytime workers (OR: 1.456; 95% CI: 1.089–1.946).^28^ Additional studies have similarly demonstrated higher serum vitamin D concentrations among outdoor workers than among indoor workers.^29^

 Although dietary sources contribute to vitamin D intake, diet is generally not a major determinant of circulating 25(OH)D levels. Dietary intake was not assessed during the present intervention; however, previous research has documented inadequate vitamin D intake across diverse populations. In a cross-sectional study of 1,222 adults, dietary inadequacy reached 100% among both males and females, with a median intake of 3.4 μg/day, less than half the Estimated Average Requirement.^30^ These findings suggest that dietary intake alone is unlikely to substantially influence 25(OH)D concentrations, particularly in regions with abundant sunlight. 

 Although no causal effect of vitamin D supplementation on adiposity was identified, individuals with excess weight consistently exhibit lower vitamin D concentrations. Monitoring vitamin D status in overweight individuals and promoting adequate sun exposure, healthy dietary habits, and regular physical activity may help improve vitamin D status and reduce the adverse effects of excess adiposity on this parameter. 

 Despite its methodological strengths, this study has several limitations. Information on sun exposure habits was not collected, preventing assessment of their influence on 25(OH)D concentrations. However, the absence of increased 25(OH)D levels in the placebo group suggests limited variation in sun exposure within this relatively homogeneous population. Future studies should incorporate assessments of sun exposure and dietary intake and use advanced body composition techniques such as DXA and MRI to further clarify the relationship between vitamin D and adiposity. 

 Furthermore, a substantial number of participants were excluded after randomization because baseline vitamin D concentrations exceeded 30 ng/mL or because of incomplete data, resulting in a per-protocol analysis. Although both ITT and PP analyses were specified in the study protocol to reduce bias,^1^ post-randomization exclusions may have introduced attrition bias. However, restricting the analysis to participants with confirmed vitamin D insufficiency or deficiency was necessary to evaluate the metabolic effects of supplementation in the target population and remained consistent with the study objectives. 

## CONCLUSION

 No significant differences in body adiposity parameters were observed between the cholecalciferol-supplemented groups and the placebo group, despite a significant increase in plasma 25(OH)D concentrations among participants receiving vitamin D. The association between vitamin D and obesity, the underlying mechanisms of this relationship, and the increase in vitamin D levels accompanying reductions in adiposity remain incompletely understood. Furthermore, whether obesity contributes causally to low vitamin D levels or whether vitamin D deficiency promotes obesity has yet to be fully established. Additional studies are needed to clarify the relationship between vitamin D status and excess body weight in this worker population. The present findings support the hypothesis that adiposity is more likely to influence vitamin D concentrations than vitamin D status is to influence adiposity. 

## Data Availability

Data supporting the findings of this study are available from the corresponding author, Luiz Menezes Júnior, upon reasonable request.
